# Role of nutritional status and intervention in oesophageal cancer treated with definitive chemoradiotherapy: outcomes from SCOPE1

**DOI:** 10.1038/bjc.2016.129

**Published:** 2016-06-21

**Authors:** S Cox, C Powell, B Carter, C Hurt, Somnath Mukherjee, Thomas David Lewis Crosby

**Affiliations:** 1 Department of Oncology, Velindre Cancer Centre, Cardiff CF14 2TL, UK; 2 Institute of Primary Care and Public Health, Cardiff University School of Medicine, Neuadd Meirionnydd, Heath Park, Cardiff CF14 4YS, UK; 3 Wales Cancer Trials Unit, Cardiff University, Neuadd Meirionnydd, Heath Park, Cardiff CF14 4YS, UK; 4 Department of Oncology, Oxford Cancer Centre, University of Oxford, Level 2 Admin, Churchill Hospital, Oxford OX3 7LE, UK

**Keywords:** chemoradiotherapy, malnutrition, nutritional intervention, Nutritional Risk Index, oesophageal cancer, SCOPE1

## Abstract

**Background:**

Malnutrition is common in oesophageal cancer. We aimed to identify nutritional prognostic factors and survival outcomes associated with nutritional intervention in the SCOPE1 (Study of Chemoradiotherapy in OesoPhageal Cancer with or without Erbitux) trial.

**Methods:**

Two hundred and fifty eight patients were randomly allocated to definitive chemoradiotherapy (dCRT) +/− cetuximab. Nutritional Risk Index (NRI) scores were calculated; NRI<100 identified patients at risk of malnutrition. Nutritional intervention included dietary advice, oral supplementation or major intervention (enteral feeding/tube placement). Univariable and multivariable analyses using Cox proportional hazard modelling were conducted.

**Results:**

At baseline NRI<100 strongly predicted for reduced overall survival (hazard ratio (HR) 12.45, 95% CI 5.24–29.57; *P*<0.001). Nutritional intervention improved survival if provided at baseline (dietary advice (HR 0.12, *P*=0.004), oral supplementation (HR 0.13, *P*<0.001) or major intervention (HR 0.13, *P*=0.003)), but not if provided later in the treatment course. Cetuximab patients receiving major nutritional intervention had worse outcomes compared with controls (13 *vs* 28 months, *P*=0.003).

**Conclusions:**

Pre-treatment assessment and correction of malnutrition may improve survival outcomes in oesophageal cancer patients treated with dCRT. Nutritional Risk Index is a simple and objective screening tool to identify patients at risk of malnutrition.

Oesophageal cancer is the eighth most common cancer worldwide with a 5-year survival rate of <20% (Cancer Research UK, 2016). Malnutrition affects up to 80% of the patients and is multifactorial in aetiology (Riccardi and Allen, 1991). Patients often present late with obstructive symptoms, cachexia, weight loss due to locally advanced disease. The psychological impact of diagnosis can result in low mood and depression, which may further reduce appetite (Van Cutsem and Arends, 2005).

Definitive chemoradiotherapy (dCRT) is a treatment option for localised oesophageal cancer, particularly in squamous cell carcinoma or in patients with adenocarcinoma deemed unsuitable for surgery (Herskovic *et al*, 1992; Smith *et al*, 1998; Cooper *et al*, 1999; Minsky *et al*, 2002; Bedenne *et al*, 2007; Crosby *et al*, 2013). Nearly half of the patients experience grade 3–4 gastrointestinal toxicities during dCRT (Crosby *et al*, 2013) and 20% may require invasive nutritional support (Gwynne *et al*, 2011). Nutritional intervention improves weight gain, performance status, tolerability of treatment, overall survival (OS) and quality of life in oncology patients (Lee *et al*, 2016). However prospective studies specifically evaluating the impact of malnutrition and nutritional intervention in patients with oesophageal cancer treated with dCRT are lacking.

The SCOPE1 (Study of Chemoradiotherapy in OesoPhageal Cancer with or without Erbitux) trial was a randomised controlled phase II/III trial comparing cisplatin-capecitabine-based dCRT for oesophageal cancer with or without cetuximab (Crosby *et al*, 2013). Two hundred and fifty eight patients were recruited from 36 centres in the UK between February 2008 and January 2012; the trial was stopped at the phase II stage because it met criteria for futility. The study reported an OS detriment in the cetuximab arm (22.1 months (95% CI 15.1–24.5) *vs* 25.4 months (95% CI 20.5–37.9); adjusted HR 1.53 (95% CI 1.03–2.27); *P*=0.035).

The aim of this study was to identify nutritional prognostic factors and the survival outcome of nutritional interventions in patients recruited to SCOPE1. We hypothesised that poor nutritional status at baseline would be associated with worse outcomes and nutritional intervention may improve survival.

## Materials and methods

### Study design

Patients were randomised in a 1 : 1 allocation ratio. The control arm received four cycles of chemotherapy with cisplatin (60 mg m^−2^ IV day 1 of 21) and capecitabine (625 mg m^−2^ po bd, continuously). Concurrent conformal radiotherapy (50 Gy in 25 fractions over 5 weeks, 2 Gy per fraction) started with cycle 3 (week 7). In the research arm, cetuximab was commenced with cycle 1 (400 mg m^−2^ day 1 of week 1, then 250 mg m^−2^ weekly thereafter for 11 weeks).

The full trial protocol has been published elsewhere and was approved by the UK Medicines and Healthcare Products Regulatory Agency and a multicentre research ethics committee (Hurt *et al*, 2011). The SCOPE1 trial was an International Standard Randomised Controlled Trial, number 47718479. Written informed consent was obtained from all recruited patients.

### Data collection

Data on the following nutritional parameters were prospectively collected at two time points, baseline and prior to dCRT (week 7): albumin (g l^−1^), body mass index (BMI, weight (kg) per height^2^ (metres)), Mellow score for dysphagia (grade 0–4) (Mellow and Pinkas, 1985), performance status (0–1) and nutritional intervention received (none, dietary advice, oral supplements or major intervention). Major intervention was defined as enteral feeding via nasogastric/nasojejunal tube placement, percutaneous endoscopic gastrostomy (PEG) or jejunostomy.

Due to difficulties in ascertaining usual body weight in cancer patients, the Lorentz formula was used to calculate ideal body weight (Bouillanne *et al*, 2005). Percentage weight loss was defined as ((current body weight−ideal body weight)/ideal body weight) × 100. The nutritional risk index (NRI) was calculated at each time point using the following formula: NRI=(1.519 × albumin g dl^−1^) + 41.7(present weight/ideal weight) (Buzby *et al*, 1988; The Veterans Affairs Total Parenteral Nutrition Cooperative Study Group, 1991; Aziz *et al*, 2011). Patients were stratified according to the risk of malnutrition: NRI score ⩾100: no risk; NRI 97.5–100: mild risk; NRI 83.5–97.5: moderate risk; NRI<83.5: major risk.

Cross tabulations of median survival were generated for all effect modifiers and compared independently with the NRI and study design stratification variables (treatment as allocated, centre, type of tumour, stage, reason for non-surgical therapy, age, gender, baseline weight and dysphagia score).

### Statistical analysis

The main analysis determined the effect modifiers of survival using a multivariable model at baseline. Cox proportion hazards regression was used to model survival. As trial participants were recruited from 36 centres, treatment centre was included as a frailty to adjust for clustering.

The baseline Cox proportion hazards model included variables consistent with the main trial analysis as *a priori* prognostic variables (centre, disease stage, reason for no surgery, tumour type (adenocarcinoma *vs* squamous histology), tumour stage, trial arm, performance status, sex, tumour length, radiation dose, cisplatin dose, capecitabine dose and age group). Additional justified effect modifiers (including biochemistry and nutritional parameters) were tested for inclusion in the base model. The main effects of these modifiers and the pre-specified interactions were sequentially introduced in order of statistical importance using a likelihood ratio test (*P*⩽0.01) independently for each time point (baseline and pre-dCRT). Parameter estimates, standard errors and *P* values were calculated. The proportional hazards assumption was assessed visually using Kaplan-Meier plots. Non-parametric log-rank tests were used to assess differences in hazard functions across subgroups.

## Results

Data from 258 patients recruited to the SCOPE1 trial were evaluated; details of the main analysis are published elsewhere (Crosby *et al*, 2013). The median length of follow-up was 25.0 (IQR 12.6-42.7) months. The number of patients in each NRI group at baseline was balanced between the two treatment arms (dCRT alone *vs* dCRT plus cetuximab, data not shown).

The majority of patients were classified not at risk of malnutrition (NRI⩾100) at baseline (217 (84%) patients); 14 (5%) were at mild risk (NRI 97.5–100), 22 (9%) at moderate risk (NRI 83.5–97.5) and only 5 (2%) were calculated to be at major risk (NRI<83.5). However, after 6 weeks of induction chemotherapy the number of patients at moderate/major malnutrition risk had increased (179 patients (70%), no risk; 16 (6%), mild risk; 48 (19%), moderate risk; 11 (4%), major risk).

The number of patients receiving nutritional intervention increased during induction chemotherapy (143 (56%) patients at baseline *vs* 192 (75%) patients prior to dCRT). Although the number of patients receiving dietary advice alone remained approximately stable (44 (17%) *vs* 40 (16%) patients), the use of oral supplements (74 (29%) *vs* 110 (43%) patients) and feeding tubes increased on treatment (25 (10%) *vs* 42 (16%) patients).

Median OS for patients according to NRI score and nutritional intervention received at baseline and prior to dCRT are shown in Tables 1 and 2. Tumour length (6–8 cm), stage III disease and receiving <50 Gy radiotherapy were identified as independent prognostic factors in multivariable analysis (*P*<0.01), (Table 3).

**Figure 1:**
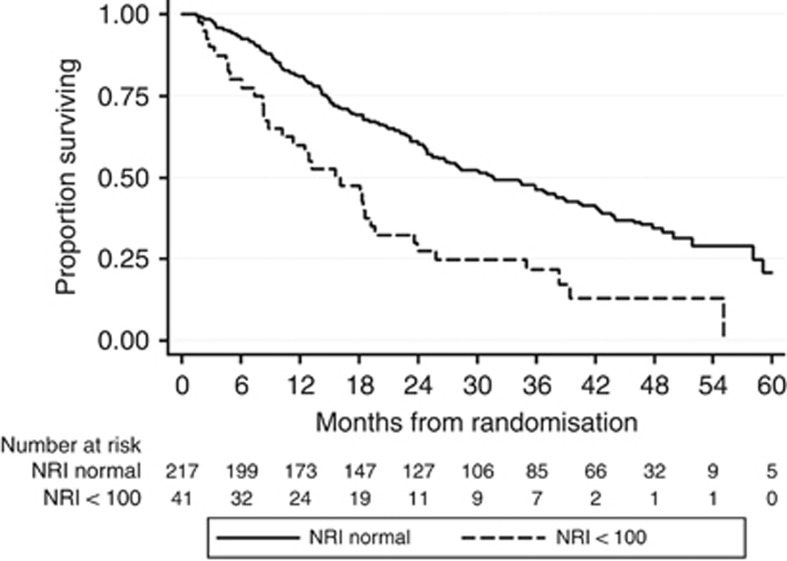
Kaplan-Meier curve of overall survival according to baseline NRI score (log-rank *P*-value <0.001). Abbreviation: NRI, nutritional risk index.

**Figure 2:**
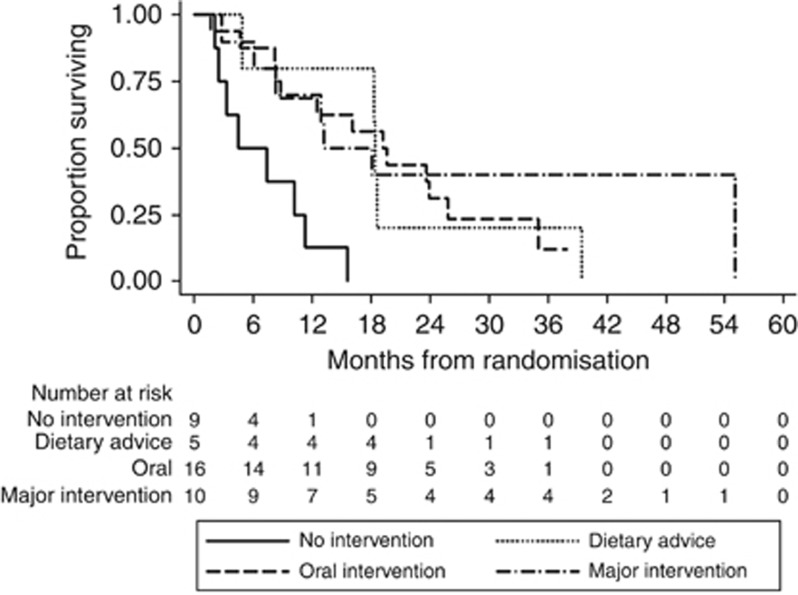
Kaplan-Meier curve of overall survival for patients with baseline NRI<100 according to nutritional intervention received at baseline (log-rank *P*=0.001). Abbreviation: NRI, nutritional risk index.

Overall survival was significantly worse in patients classified to be at the risk of malnutrition at baseline (NRI<100, median survival time 15.7 months; IQR 7.4–25.8) compared with patients not at risk (NRI⩾100, median survival time 31.6 months; IQR 14.7–58.0) (HR 12.5 (95% CI 5.2–29.6), *P*<0.001) (Figure 1). In these at-risk patients, providing nutritional intervention at baseline was associated with an improved OS (dietary advice (HR 0.12 (0.03–0.51), *P*=0.004), oral supplements (HR 0.13, (0.04–0.39), *P*<0.001) and major intervention (HR 0.13 (0.03–0.50), *P*=0.003) (Table 3; Figure 2). Univariable main effects not included in the multivariable model are shown in Supplementary Table S1.

The median survival for patients with NRI<100 and NRI⩾100 prior to starting dCRT was 15.4 and 30.3 months, respectively, but after adjustment for other factors this was not significant in the multivariable model (HR 1.79 (0.64–5.04), *P*=0.27, full model not shown). Furthermore, nutritional intervention provided to at-risk patients after the commencement of induction chemotherapy was no longer associated with a survival benefit (dietary advice alone HR 1.31, *P*=0.72; oral supplements HR 0.86, *P*=0.81; major intervention HR 0.84, *P*=0.81).

The only survival difference based on nutritional parameters between the trial arms was seen in patients who required major nutritional intervention; those allocated to dCRT plus cetuximab had a shorter OS than those treated with dCRT alone at both baseline (HR 4.7 (1.4–15.70), *P*=0.01) and pre-dCRT (HR 5.4 (1.75–16.36), *P*=0.003).

## Discussion

This study suggests that increased nutritional risk at baseline is associated with reduced survival in patients with localised oesophageal cancer treated with dCRT. An NRI score <100 strongly predicted for reduced OS. An improvement in survival was observed following baseline nutritional intervention with dietary advice, oral supplementation or major intervention. A similar benefit was not observed if nutritional intervention occurred later in the treatment course.

To our knowledge this is the first evaluation of prospectively collected data to demonstrate the benefit of nutritional intervention in oesophageal cancer. Other groups have identified prognostic nutritional factors in patients treated with dCRT. Thomas *et al* (2004) performed recursive partitioning analysis of pre-treatment variables in 416 patients; only pre-treatment weight loss >10% in the 6 months prior to treatment was identified as a significant factor. BMI>18 kg m^−2^, Atkinson dysphagia score *<*2, dose of RT>50 Gy and complete response to CRT were found to be independent prognostic factors for survival in a retrospective analysis of 105 patients (Di Fiore *et al*, 2007). In a more recent study by the same group, OS was 25 months in patients with persistent malnutrition compared with 42 months in those who remained well nourished during CRT (Di Fiore *et al*, 2014). There is also evidence for nutritional factors as predictors of benefit following dCRT. Di Fiore *et al* (2007) found a significant difference in baseline percentage weight loss, albumin level and BMI between responders and non-responders; response rates to dCRT were significantly lower in patients with malnutrition at baseline and during treatment.

Malnutrition in patients treated for oesophageal cancer is common and may be related to the disease or its treatment (Muscaritoli *et al*, 2011). Appropriate nutritional support is important for maintaining treatment intensity and may influence outcome (Lee *et al*, 2016). A formalised nutritional pathway for patients receiving dCRT significantly reduced complications with less weight loss, fewer unplanned hospital admissions and greater radiotherapy completion rates observed (Odelli *et al*, 2005). Concerns regarding the use of PEG feeding tubes in oesophageal cancer relating to safety of dilatation and potential for inoculation metastasis have been raised (Singh and Gelrud, 2015). However in a retrospective analysis, PEG placement was successful in the majority of patients prior to multimodality treatment for oesophageal cancer and was significantly related to attainment of target doses of chemoradiotherapy (*P*=0.034), and survival at 12 months (*P*=0.02) (Margolis *et al*, 2003). In our study, patients with NRI⩾100 at baseline requiring major nutritional intervention had a worse outcome than those who required no nutritional support (24.7 *vs* 34.5 months, Table 1). This may represent patients with significant dysphagia at presentation due to more locally advanced disease and subsequent reduced survival rates. For patients with mild (NRI 97.5–100) or moderate/major (NRI<97.5) risk, major nutritional intervention improved survival (7.4 *vs* 44 months and 3.5 *vs* 10.6 months, respectively, Table 1).

Patients allocated to cetuximab arm who required a major nutritional intervention at baseline had worse survival rates compared with controls (13.3 *vs* 28 months); a similar finding was observed if nutritional intervention occurred prior to dCRT (13.3 *vs* 31.1 months). The cause for this survival difference is unclear and based on small patient numbers, however, one hypothesis is that fewer patients receiving cetuximab completed the standard protocol treatment, with significant differences in the number of chemotherapy cycles received and total radiotherapy dose delivered (Crosby *et al*, 2013).

Our study has limitations; first, only 16% of the patients were deemed at risk of malnutrition at baseline, which is lower than anticipated. This may represent selection bias as only patients with performance status 0–1 were recruited, or an inadequacy in dietetic screening as formal dietetic assessment was not a trial prerequisite. Second, although the nutritional data were collected prospectively, the analysis itself is retrospective and requires further prospective validation. Third, although data on whether patients received nutritional intervention were collected, the details of the intervention, intensity of dietetic follow-up or variation between centres were not collected. A randomised control trial of ‘conventional hospital protocol nutritional intervention' *vs* ‘NRI-directed nutritional intervention' may be required to assess the true value of NRI-directed intervention in this patient group.

In conclusion, assessment and correction of poor nutritional state at baseline may be a simple and cost-effective intervention that improves survival outcomes in oesophageal cancer patients treated with dCRT. Nutritional Risk Index serves as a simple and objective screening tool to identify patients at risk of malnutrition. In this study, the benefit of nutritional intervention was no longer observed once treatment had commenced, highlighting the need for early nutritional assessment and intervention.

## Figures and Tables

**Table 1:** Median survival time (months) according to maximum nutritional intervention received and nutritional risk index (NRI) score at baseline

	**NRI score**			
	**⩾100 (*****N***)	**>97.5 (*****N***)	**<97.5 (*****N***)	**All patients (*****N***)^a^
None	34.5 (105)	7.4 (3)	3.5 (6)	29.2 (114)
Dietary advice	31.6 (39)	28.8 (2)	18.4 (3)	28.9 (44)
Oral supplements	24.6 (58)	23.9 (5)	19.1 (11)	23.4 (74)
Major nutritional intervention	24.7 (15)	44.0 (4)	10.6 (6)	23.6 (25)
All patients	27.9 (217)	21.1 (14)	11.3 (21)	24.9 (257)

a Data on nutritional intervention received at baseline were missing for one patient.

**Table 2:** Median survival time (months) according to maximum nutritional intervention received and nutritional risk index (NRI) score prior to definitive chemoradiotherapy (dCRT)

	**NRI score**			
	**⩾100 (*****N***)	**>97.5 (*****N***)	**<97.5 (*****N***)	**All patients (*****N***)^a^
None	35.7 (56)	23.1 (9)	11.3 (1)	35.1 (66)
Dietary advice	36.7 (29)	12.2 (10)		35.9 (39)
Oral supplements	24.8 (74)	19.1 (29)	11.3 (6)	23.2 (109)
Major nutritional intervention	24.7 (20)	16.5 (16)	13.0 (4)	21.4 (40)
All patients	30.2 (179)	18.3 (64)	12.5 (11)	24.9 (254)

a Data on NRI score prior to dCRT were missing for four patients.

**Table 3:** Univariable and multivariable analysis of baseline prognostic factors of overall survival

	**Survival**	**Univariable analysis**		**Multivariable analysis**	
	***N*****, median (Q3-Q1)**	**HR, 95% CI**	***P*****-value**	**HR, 95% CI**	***P*****-value**
Age					
<70 years	160, 26.2 (43–14.3)	Reference		Reference	
⩾70 years	98, 22.6 (40.3–9.2)	1.33, (0.98–1.81)	0.068	1.19, (0.82–1.72)	0.366
Gender					
Male	145, 24 (38.6–11.3)	1.47, (1.07–2.00)	0.016	1.38, (0.95–2.02)	0.095
Female	113, 26.1 (46.9–14.3)	Reference		Reference	
Performance status					
0	131, 27.2 (44–14.7)	Reference		Reference	
1	127, 24.5 (40.3–10)	1.17, (0.86–1.59)	0.320	0.96, (0.67–1.39)	0.833
Tumour length					
<2 cm	56, 30.7 (46.4–12.1)	Reference		Reference	
2–4 cm	85, 30.3 (46.9–14.8)	1.00, (0.63–1.57)	0.992	1.16, (0.70–1.89)	0.568
4–6 cm	55, 24.9 (42.4–11.5)	1.48, (0.93–2.36)	0.102	1.40, (0.79–2.47)	0.250
6–8 cm	62, 18.2 (35.9–10)	1.87, (1.18–2.96)	0.008	1.81, (1.05–3.12)	0.034
Stage					
I+II	103, 35.9 (46.6–15.3)	Reference		Reference	
III	155, 23.2 (37–11.3)	1.66, (1.20–2.30)	0.002	1.58, (1.05–2.38)	0.027
Tumour type					
Squamous cell	188, 25.4 (43.3–13.6)	Reference		Reference	
Adenocarcinoma	70, 23.2 (39.1–10.2)	1.28, (0.92–1.78)	0.144	0.97, (0.62–1.52)	0.907
Reason for no surgery					
Patient choice	97, 26.7 (46.6–14.7)	Reference		Reference	
Comorbidity	36, 31.6 (42.7–11.1)	1.24, (0.79–1.94)	0.350	0.93, (0.51–1.70)	0.817
Local extent	122, 24 (40.6–11.5)	1.20, (0.86–1.69)	0.285	0.87, (0.58–1.29)	0.478
Treatment arm					
dCRT only	129, 23.3 (39.4–10.2)			Reference	
dCRT + cetuximab	129, 27.8 (46–14.8)	1.27, (0.94–1.71)	0.125	0.82, (0.49–1.37)	0.440
Full radiation protocol dose					
Yes	217, 30.1 (46–14.9)	Reference		Reference	
No	41, 8.2 (20.8–2.9)	3.46, (2.36–5.07)	<0.001	2.92, (1.49–5.75)	0.002
% of full cisplatin protocol dose					
⩾95%	106, 35.2 (46.9–16.9)	Reference		Reference	
⩾75–<95%	76, 29 (44.3–15.3)	1.29, (0.88–1.90)	0.184	1.11, (0.70–1.75)	0.672
⩾50–<75%	41, 18.4 (36–12.5)	2.20, (1.44–3.38)	<0.001	1.76, (1.00–3.13)	0.051
<50%	35, 10.2 (24.7–5.9)	3.17, (2.00, 5.03)	<0.001	1.80, (0.86–3.76)	0.118
% of full capecitabine protocol dose					
⩾95%	82, 32.8 (45.3–14.9)	Reference		Reference	
⩾75–<95%	90, 28.1 (45.9–14.8)	1.07, (0.73–1.59)	0.719	0.99, (0.62–1.58)	0.956
⩾50–<75%	52, 22.5 (43.3–7.7)	1.58, (1.03–2.41)	0.035	0.97, (0.54–1.73)	0.907
<50%	34, 15.6 (23.2–5.9)	2.34, (1.46–3.76)	<0.001	0.73, (0.32–1.69)	0.465
NRI					
⩾100	217, 28 (45.9–14.2)	Reference		Reference	
<100	41, 15.6 (24.5–8)	2.26, (1.54–3.30)	<0.001	12.45, (5.24–29.6)	<0.001
Nutritional intervention					
None	114, 29.2 (46.8–12.8)			Reference	
Dietary advice alone	44, 29 (44.6–15.7)	1.05, (0.67–1.63)	0.835	1.18, (0.59–2.39)	0.638
Oral supplements	74, 23.4 (35.8–10.9)	1.49, (1.04–2.15)	0.030	1.00, (0.54–1.85)	0.992
Major intervention	25, 23.7 (40.3–10)	1.20, (0.69–2.08)	0.525	0.53, (0.19–1.50)	0.232
Nutritional intervention in the CRT + cetuximab group					
None				Reference	
Dietary advice alone				1.19, (0.45–3.15)	0.720
Oral supplements				1.94, (0.89–4.20)	0.093
Major intervention				4.69, (1.40–15.7)	0.012
Nutritional intervention in those with a baseline NRI<100					
None				Reference	
Dietary advice alone				0.12, (0.03–0.51)	0.004
Oral supplements				0.13, (0.04–0.39)	<0.001
Major intervention				0.13, (0.03–0.50)	0.003

Abbreviations: CI=confidence interval; CRT=chemoradiotherapy; dCRT=definitive chemoradiotherapy; HR=hazard ratio; NRI=nutritional risk index.
