# Supplementary material for: Role of nutritional status and intervention in oesophageal cancer treated with definitive chemoradiotherapy: outcomes from SCOPE1
Source: Br J Cancer. 2016 Jun 21;115(2):172–7. doi: 10.1038/bjc.2016.129 (PMC4947693; doi:10.1038/bjc.2016.129)
Supplement: Supplementary Information [file bjc2016129x1.doc]

**Supplementary Material**

| Supplementary Table S1 – Univariable analysis of the proposed baseline main effect risk modifiers | | | | |
| --- | --- | --- | --- | --- |
|  |  | **Survival** | **Univariable Analysis** | |
|  |  | ***N*, Median (Q3-Q1)** | **HR, 95% CI** | ***p*** |
| **Nutritional parameters** | | | | |
| Weight loss at baseline, compared to ideal weight | No more than 5% | 208, 27 (43.8 - 13.6) | Reference | |
|  | 5 to 10% | 16, 22.7 (40 - 10.4) | 1.53 (0.83-2.72) | 0.148 |
|  | More than 10% | 30, 14.8 (35.8 - 8.3) | 1.84 (1.19-2.86) | 0.006 |
| BMI | Less 18.5 | 12, 14.2 (28 - 7.2) | 1.70 (0.84-3.44) | 0.143 |
|  | 18.5 to 25 | 116, 25.9 (43.1 - 11) | Reference | |
|  | Above 25 | 130, 25.2 (42.4 - 13.3) | 0.87 (0.64-1.19) | 0.394 |
| Dysphagia score | < 3 | 226, 25.4 (42.4 - 13.3) | Reference | |
|  | ≥ 3 | 32, 21.3 (41.7 - 9.5) | 1.34 (0.87-2.08) | 0.182 |
| **Biochemical parameters** | | | | |
| Serum albumin | <35g/L | 18, 12.1 (24.5 - 8.8) | 2.30 (1.34-3.95) | 0.002 |
|  | ≥ 35g/L | 240, 25.9 (43.8 - 13.6) | Reference | |
| Haemoglobin | <12g/dL | 37, 23.2 (35.9 - 8.4) | 1.15 (0.75-1.78) | 0.518 |
|  | ≥ 12g/dL | 221, 25.4 (44.0 – 13.3) | Reference | |
| Phosphate | <130IU/L | 183, 28.0 (14.0-14.0) | Reference | |
|  | ≥ 130IU/L | 75, (21.6 (36.9-9.3) | 1.30 (0.94-1.81) | 0.116 |
